# Metabolic syndrome traits exhibit genotype-by-environment interaction in relation to socioeconomic status in the Mexican American family heart study

**DOI:** 10.3389/fgene.2024.1240462

**Published:** 2024-03-01

**Authors:** Vincent P. Diego, Eron G. Manusov, Xi Mao, Marcio Almeida, Juan M. Peralta, Joanne E. Curran, Michael C. Mahaney, Harald Göring, John Blangero, Sarah Williams-Blangero

**Affiliations:** ^1^ South Texas Diabetes and Obesity Institute, School of Medicine, University of Texas Rio Grande Valley, Brownsville, TX, United States; ^2^ Department of Human Genetics, School of Medicine, University of Texas Rio Grande Valley, Brownsville, TX, United States; ^3^ Department of Economics, University of Texas Rio Grande Valley, Brownsville, TX, United States

**Keywords:** socioeconomic status, metabolic syndrome, GxE, Mexican Americans, genotype-phenotype network

## Abstract

**Background:** Socioeconomic Status (SES) is a potent environmental determinant of health. To our knowledge, no assessment of genotype-environment interaction has been conducted to consider the joint effects of socioeconomic status and genetics on risk for metabolic disease. We analyzed data from the Mexican American Family Studies (MAFS) to evaluate the hypothesis that genotype-by-environment interaction (GxE) is an essential determinant of variation in risk factors for metabolic syndrome (MS).

**Methods:** We employed a maximum likelihood estimation of the decomposition of variance components to detect GxE interaction. After excluding individuals with diabetes and individuals on medication for diabetes, hypertension, or dyslipidemia, we analyzed 12 MS risk factors: fasting glucose (FG), fasting insulin (FI), 2-h glucose (2G), 2-h insulin (2I), body mass index (BMI), waist circumference (WC), leptin (LP), high-density lipoprotein-cholesterol (HDL-C), triglycerides (TG), total serum cholesterol (TSC), systolic blood pressure (SBP), and diastolic blood pressure (DBP). Our SES variable used a combined score of Duncan’s socioeconomic index and education years. Heterogeneity in the additive genetic variance across the SES continuum and a departure from unity in the genetic correlation coefficient were taken as evidence of GxE interaction. Hypothesis tests were conducted using standard likelihood ratio tests.

**Results:** We found evidence of GxE for fasting glucose, 2-h glucose, 2-h insulin, BMI, and triglycerides. The genetic effects underlying the insulin/glucose metabolism component of MS are upregulated at the lower end of the SES spectrum. We also determined that the household variance for systolic blood pressure decreased with increasing SES.

**Conclusion:** These results show a significant change in the GxE interaction underlying the major components of MS in response to changes in socioeconomic status. Further mRNA sequencing studies will identify genes and canonical gene pathways to support our molecular-level hypotheses.

## Contribution to research

Epidemiologic studies have identified socioeconomic status as a potent social determinant of health. Poor access to healthcare, lack of education, inadequate financial resources, experience with violence, and constant vigilance associated with low socioeconomic status explain increased morbidity and mortality in populations that live in poverty. Genotype-by-environment interaction (GxE) studies detect if genetic factors interact with the environment to produce phenotypic variation. Our results provide evidence of interactions between SES and metabolic syndrome at a molecular level in Mexican Americans living in South Texas.

Socioeconomic status (SES) is a composite measure of economic and social status that incorporates education, income, and occupation elements. SES influences health through the socialization of positive health habits, education, and the ability to access healthcare resources (Marmot et al., 1991; Marmot, 2003a; Marmot, 2003b; Marmot, 2015; Pillay-van Wyk and Bradshaw, 2017; Rosengren et al., 2019; Averbuch et al., 2022). SES is inversely correlated to disease-specific morbidity/mortality risk for cardiovascular disease, metabolic syndrome, hypertension, diabetes, frailty, and obesity (Chandola et al., 2007; Bijlsma-Rutte et al., 2018; Conway et al., 2018; Allegrini et al., 2020; Manusov et al., 2021; Diego et al., 2023). The SES effects on health are even more prominent in Latino populations, especially Mexican Americans, where a higher prevalence of obesity, diabetes, hypertension, and hyperlipidemia are observed (Hazuda et al., 1991; Heidi Ullmann et al., 2011; Hostinar et al., 2017a; Conway et al., 2018; Diego et al., 2023). An estimated 35 percent of Mexican Americans meet the American Heart Association criteria for metabolic syndrome (MS defined as high triglycerides (>150 mg/dL or on cholesterol medicine, High-Density Lipoprotein (HDL) < 40 mg/dL men and 50 mg/dL women, blood pressure <135/85 or on medications, fasting blood glucose> 100 mg/dL) (Beltrán-Sánchez et al., 2016; Manusov et al., 2019). The prevalence of MS is higher in certain Mexican American groups (older adults, women, non-recent immigrants, US South) (Fernández-Rhodes et al., 2021).

There is an inverse statistical correlation between SES measures and metabolic syndrome (MS) and all-cause mortality in Mexican Americans living in south Texas (Hazuda et al., 1991; Bustamante-Villagómez et al., 2021; He et al., 2021). The effects of SES are traditionally explained by social advantage/deprivation, social mobility, chronic generational poverty, neighborhood deprivation, early-life education health habits (exercise, healthy eating, sleep, quality of life), immigration, vulnerability to environmental stressors, and acculturation (Hazuda et al., 1988; Wei et al., 1996; Stafford and Marmot, 2003; Riosmena and Dennis, 2012; Diego et al., 2015; Kelly and Ismail, 2015; Khambaty et al., 2020; McDoom et al., 2020).

Significant GxE interactions exist between modifiable environmental risk factors and genes that influence cardiovascular risk, (Diego et al., 2023), hyperlipidemia, obesity, and non-alcoholic fatty liver disease (sedentary lifestyle, smoking, and hormonal imbalance) (Hazuda et al., 1988; Heidi Ullmann et al., 2011; Arya et al., 2018; Dugravot et al., 2020; Muscatell et al., 2020; Ciciurkaite, 2021; Manusov et al., 2022).

Recent statistical advances in the evaluation of GxE interaction are shedding light on possible mechanisms by which genes respond to environmental stressors. Specific genes have been determined to play roles in lipid metabolism, inflammation, and insulin regulation, with pro-inflammatory pathways also influencing obesity development. Several studies have examined the effect of gene-environment interactions for loci regulating inflammatory pathways. Environmental factors may modify risk through epigenetic or transcriptional control mechanisms or by affecting downstream regulation of metabolic pathways (Muscatell et al., 2020).

We hypothesize that environmental factors (defined by Duncan’s Socioeconomic Index, educational level, and income) influence the expression of genes related to risk for metabolic syndrome. We investigate the interaction between the SES environment and MS risk factors using a genotype-by-environment interaction (GxE) approach (Blangero et al., 2013). GxE describes how the genetic architecture underlying a given trait (MS risk factor) responds to or depends on environmental changes (SES). Whereas epidemiological studies have demonstrated the inverse association of SES with MS-related morbidity and mortality, GxE analyses can provide information on how genes interact with the environment. To formally evaluate the hypothesis that the genes underlying MS risk factors exhibit GxE interaction effects, we examined potential GxE interaction using variance components models and likelihood-based statistical inference in the phenotypic expression of metabolic syndrome in data from the Mexican American Family Studies (MAFS) (Blangero et al., 2013; Diego et al., 2023). MAFS was initially designed to characterize the genetic determinants of risk for cardiovascular disease (CVD) in Mexican Americans of South Texas. The MAFS is a large, longitudinal pedigree-based study of genetic determinants of risk for complex disease in Mexican Americans, which was initiated in 1992 (Hazuda et al., 1991).

## Methods

The Institutional Review Board at the University of Texas Health Science Center at San Antonio approved the MAFS study protocols. All study participants provided written informed consent.

### Study population

The MAFS population comprises extended Mexican American families that were randomly ascertained for studies of CVD. As described by our team in earlier publications (Hazuda et al., 1988; Hazuda et al., 1991), the initial data were collected between January 1992 and June 1995. There were 1,431 individuals recruited, belonging to 42 extended families. The probands were recruited from a single census tract of a low-income San Antonio, Texas neighborhood. Inclusion criteria included proband age between 40 years and 60 years, a spouse who was willing to participate in the study, and at least six relatives (first-, second-, and third-degree adult relatives of the proband and of the proband’s spouse) available to participate in the study. Phenotypic assessments, biochemical methods, and interviews are described in detail in previous publications (Bethony et al., 2002; Diego et al., 2003). Surveys determined social, behavioral, and lifestyle factors related to cardiovascular risk, including past medical history, educational background, household income level, reproductive history, and smoking and alcohol use. Established questionnaires assessed dietary intake and physical activity (Hazuda et al., 1988).

### Phenotypic assessments

We analyzed 12 traits that are traditional MS risk factors (fasting glucose (mg/d), fasting insulin (u/mL), 2-h glucose (mg/dL), leptin (ng/mL), high-density lipoprotein-cholesterol (mg/dL), triglycerides (mg/dL), total serum cholesterol (mg/dL), 2-h insulin (u/mL), body mass index (kg/m^2^), waist circumference (cm), systolic blood pressure (mmHg), diastolic blood pressure (mmHg))—the protocols describing how the above traits were measured referenced in earlier reports by our group (Stern et al., 1984; Hazuda et al., 1988; Haffner et al., 1989; Hazuda et al., 1991). Metabolic syndrome is distinguished from other health conditions, especially diabetes and lipid metabolism disorders, by specific criteria. These criteria include central obesity, elevated blood pressure, fasting glucose levels, triglycerides, and low HDL cholesterol. Exclusions are in place to prevent individuals with established diabetes or primary lipid disorders from being diagnosed with metabolic syndrome, ensuring clear and reproducible diagnoses in clinical practice. This approach is grounded in medical literature and helps clinicians identify individuals at higher risk for cardiovascular diseases and type 2 diabetes. To account for the impact of altered metabolism due to disease or medication, we excluded 291 individuals with diabetes, diabetes medications, hypertension, or dyslipidemia. This procedure decreased the sample upper limit to 1,140 individuals.

### Interview data

We obtained social, medical history, behavioral, and dietary data (DM status, DM medications, HTN medications, dyslipidemia medications, smoking history, leisure time, physical activity-work, total physical activity, and dietary intake including estimates of protein (g/d), carb (g/d), saturated fat (g/d), monosaturated fat (g/d), polyunsaturated fat (g/d), cholesterol (g/d), sucrose (g/d), and alcohol (g/d) consumption. To measure socioeconomic status, we used a combination of occupation, income, and education in our SES score (Diego et al., 2023). Occupations were coded, and their corresponding Duncan socioeconomic index (SEI) scores were computed as described earlier. To calculate a standardized SES (zSES) score, we standardized the sum of the standardized SEI and education variables. This variable was used as our index of the SES environment in all our GxE analyses.

We also obtained information on categorical medical history variables, physical activity, measured in units of metabolic-equivalent-tasks (METs) (Stanford 7-Day Physical Activity Recall Instrument), and data on dietary intake variables as assessed by a food frequency questionnaire developed specifically for the Mexican American population of San Antonio. All physical activity and dietary intake variables were standardized.

### Statistical analyses

We used a generalized linear mixed model **to include fixed (linear relationship to the dependent variable) and random effects** (accounting for the variability in the data arising from a hierarchical structure). We used Bayesian model selection (BMS) to account for model uncertainty and selection bias and select the most supported model (i.e., the most supported traits). (Diego et al., 2023). For all BMS analyses, we analyzed the effects of the following covariates: age, sex, age-squared, sex-by-age, sex-by-age-squared, smoking, blood alcohol, leisure physical activity, work physical activity, total physical activity, protein, carbohydrates, saturated fat, monosaturated fat, poly-saturated fat, cholesterol, sucrose, and alcohol intake. Under each trait-wise most-supported model, residuals were obtained after fitting the model and were then normalized using an inverse normalization transformation (Almasy and Blangero, 2010).

We calculated heritability, household effect, and GxE interaction effects under a maximum likelihood estimation variance components model, as implemented in SOLAR (http://solar-eclipse-genetics.org/brief-overview.html). Heritability, defined as the ratio of the additive genetic variance ( $\sigma_{g}^{2}$ ) to the total phenotypic variance ( $\sigma_{p}^{2}$ ), was estimated under a polygenic model, which posits the decomposition of $\sigma_{p}^{2}$ as a sum of $\sigma_{g}^{2}$ and the residual environmental variance ( $\sigma_{e}^{2}$ ): $\sigma_{p}^{2}=\sigma_{g}^{2}+\sigma_{e}^{2}$ (model 1). We also used a model designed to test the importance of random household effects.48–50 (Bethony et al., 2001; Bethony et al., 2002; Bethony et al., 2004) Under this model, in addition to estimating $\sigma_{g}^{2}$ , we also estimate a household variance component, denoted by $\sigma_{c}^{2}$ . The total phenotypic variance is given as $\sigma_{p}^{2}=\sigma_{g}^{2}+\sigma_{c}^{2}+\sigma_{e}^{2}$ (model 2). We first tested if the traits were significantly heritable. We then tested if $\sigma_{c}^{2}$ was significant. If $\sigma_{c}^{2}$ was not significant, then heritability was estimated under model 1, and if $\sigma_{c}^{2}$ was significant, then heritability was estimated under model 2.

There is no GxE if $\sigma_{g}^{2}$ is homogeneous across environments, and the genetic correlation, denoted by $\rho_{g}$ , equals 1 across environments (Diego et al., 2003; Almasy and Blangero, 2010). These considerations supply two distinct null sub-hypotheses of GxE. Rejecting one or both is sufficient to reject the null hypothesis of no GxE. The two criteria together amount to a definition of GxE as covariance heterogeneity across the environmental continuum. To be able to test the null hypothesis of no GxE across the continuous SES environment, $\sigma_{g}^{2}$ and $\rho_{g}$ are parameterized as functions of zSES:

$$\sigma_{g}^{2}=\exp\left[\alpha_{g}+\gamma_{g}\left({\text{zSES}}_{i}\right)\right];$$ (1)

$$\rho_{g}=\exp\left(-\lambda_{g}\left|{\text{zSES}}_{i}-{\text{zSES}}_{j}\right|\right),$$ (2)

where $\sigma_{g}^{2}$ is parameterized as an exponential function of zSES to ensure positivity and $\rho_{g}$ as an exponential decay function of pair-wise differences in zSES, i and j index individuals in the sample and $\alpha_{g}$ , $\gamma_{g}$ , and $\lambda_{g}$ are parameters to be estimated. (Stern et al., 1984; Haffner et al., 1989). The null sub-hypotheses of homogeneous $\sigma_{g}^{2}$ and $\rho_{g}$ equal to 1 are respectively given by $\gamma_{g}=0$ and $\lambda_{g}=0$ . To prevent bias in the estimation of the genetic functions, we also parameterized $\sigma_{e}^{2}$ as an exponential function in the same way as for $\sigma_{g}^{2}$ :

$$\sigma_{e}^{2}=\exp\left[\alpha_{e}+\gamma_{e}\left({\text{zSES}}_{i}\right)\right].$$ (3)

There can be no corresponding residual environmental correlation function because of the standard statistical genetic assumption of completely uncorrelated genetic and residual environmental effects. If $\sigma_{c}^{2}$ it was found to be significant under the household-effects analysis, we added variance and correlation functions for the household effects in the full GxE model:

$$\sigma_{c}^{2}=\exp\left[\alpha_{c}+\gamma_{c}\left({\text{zSES}}_{i}\right)\right];$$ (4)

$$\rho_{c}=\exp\left(-\lambda_{c}\left|{\text{zSES}}_{i}-{\text{zSES}}_{j}\right|\right).$$ (5)

We implemented a three-stage hypothesis-testing procedure. In the first stage, we tested if the traits were heritable under model 1 or model 2 as appropriate. In the second stage, for the heritable traits, we tested whether the GxE model was superior to model 1 or model 2 as appropriate. Traits for which the GxE model was superior were then further evaluated at the last stage, in which we tested the specific GSI hypotheses of $\gamma_{g}=0$ and $\lambda_{g}=0$ . At this stage, we also tested for $\gamma_{e}=0$ , $\gamma_{c}=0$ and $\lambda_{c}=0$ because the corresponding null hypotheses of homogeneous $\sigma_{e}^{2}$ , homogeneous $\sigma_{c}^{2}$ , and $\rho_{c}=1$ , respectively, are interesting in their own right. All hypotheses were tested by likelihood ratio tests, as previously described (Almasy and Blangero, 2010).

To determine the potential for dietary variables or alcohol to be a mediator of the SES variable, we performed association analyses in relation to the SES variable. In each case, the SES variable was the dependent variable and the covariate in question (one at a time) was the independent predictor variable in a simple regression model. The reported *p*-values are adjusted for multiple hypothesis testing (Supplementary Table S1) There is no evidence of the dietary or alcohol variables acting as mediators of the SES variable.

A comprehensive background on the statistical methods used for analyzing genotype-by-environment (GxE) interactions including a detailed explanation of each statistical variable, its relevance, and significance in the context of GxE research is included in the Supplementary Material.

## Results

The ages of individuals in our sample range from 18 to 94 years of age, with a mean age of 35 years. The descriptive statistics for the MS trait data are presented in Table 1. Missing data were not imputed. Based on an established definition of MS the prevalence of MS (Engin, 2017) in our total sample of 1,140 individuals is 27.21%.

**TABLE 1:** Descriptive statistics of metabolic syndrome traits.

Trait	Sample size			Mean			Standard deviation		
	All	Males	Females	All	Males	Females	All	Males	Females
Fasting glucose mg/dL	1,031	441	590	86.51	88.25	85.21	10.55	11.49	9.57
Fasting insulin u/mL	1,013	432	581	13.66	13.76	13.59	14.66	17.91	12.19
2-h glucose mg/dL	1,009	430	579	100.22	94.98	104.12	31.37	31.81	30.46
2-h insulin u/mL	983	419	564	73.48	60.67	82.99	68.96	64.93	70.31
Body mass index kg/m^2^	1,034	441	593	28.56	27.83	29.10	6.27	5.67	6.62
Waist circumference cm	913	440	590	92.23	94.20	90.76	15.95	14.57	16.75
Leptin ng/mL	962	411	551	10.10	4.94	13.94	8.68	5.10	8.81
Total serum cholesterol mg/dL	1,028	440	589	185.87	185.73	185.98	38.40	38.38	38.41
HDL-C mg/dL	1,028	440	588	50.54	48.04	52.40	12.70	13.05	12.10
Triglycerides mg/dL	1,029	440	589	137.37	147.31	129.94	125.05	112.24	133.34
Systolic blood pressure mmHg	1,032	441	591	116.29	119.88	113.61	14.68	13.51	14.94
Diastolic blood pressure mmHg	1,032	441	591	69.87	71.85	68.39	9.77	9.39	9.79

In Table 2, we present the heritability estimates for each of the MS traits, along with the covariates included in the BMS most-supported model and the percent variance they explained. Only four traits (waist circumference, total serum cholesterol, systolic blood pressure, and diastolic blood pressure) showed evidence of a significant household variance component (Table 2).

**TABLE 2:** Heritability of and household effects in metabolic syndrome traits.

Trait	N^a^	h^2^ (SE)^a^ *p*-value	c^2^ (SE)^b^ *p*-value	Significant covariates^‡^	Variance accounted for by covariates
Fasting glucose	1,031	0.45 (0.06) *p* = 6.8E-20	-----	Age, sex	0.08
Fasting insulin	1,013	0.45 (0.07) *p* = 4.1E-18	-----	none	NA
2-h glucose	1,009	0.36 (0.06) *p* = 8.0E-13	-----	Age, sex, smoke	0.12
2-h insulin	983	0.28 (0.06) *p* = 6.1E-10	-----	Age, sex	0.05
Body mass index	1,034	0.53 (0.06) *p* = 3.6E-24	-----	Age, age-squared	0.06
Waist circumference	913	0.43 (0.07) *p* = 1.6E-09	0.9 (0.05) *p* = 0.04	Age, age-squared, SFAT, MFAT	0.10
Leptin	962	0.34 (0.07) *p* = 6.1E-10	-----	Age, sex	0.25
Total serum cholesterol	1,129	0.32 (0.07) *p* = 2.8E-09	0.12 (0.05) *p* = 0.003	Age, sex, age-squared, age-squared-by-sex	0.13
HDL-C	1,028	0.48 (0.07) *p* = 3.2E-24	-----	Sex, chol, dALCO	0.06
Triglycerides	1,029	0.45 (0.07) *p* = 7.8E-20	-----	Age	0.02
Systolic blood pressure	1,030	0.31 (0.07) *p* = 3.4E-08	0.11 (0.05) *p* = 0.01	Age, sex, age-squared, age-by-sex, dALCO	0.28
Diastolic blood pressure	1,126	0.29 (0.07) *p* = 2.0E-07	0.12 (0.05) *p* = 0.005	Age, sex, age-squared	0.14

^a^ For normalized residuals (see text).

^b^ Ratio of household variance to the total phenotypic variance.

Test results of the GxE model *versus* model 1 or model 2, corrected for multiple hypothesis testing, are presented in Table 3. Five traits showed the potential for GxE effects (fasting glucose, 2-h glucose, total serum cholesterol, systolic blood pressure, and diastolic blood pressure). We note that only systolic blood pressure has household variance and correlation functions added to the GxE model (Table 4).

**TABLE 3:** Evidence for genotype-by-SES interaction.

Trait	*p*-value^a^
Fasting glucose	0.02856
Fasting insulin	1.00000
2-h Glucose	9.816E-05
2-h Insulin	0.05172
Body mass index	0.06036
Waist circumference	1.00000
Leptin	1.00000
Total serum cholesterol	3.6E-19
High density lipoprotein cholesterol	1.00000
Triglycerides	0.20208
Systolic blood pressure	0.006
Diastolic blood pressure	6.6E-18

^a^ All *p*-values adjusted or corrected for multiple hypothesis testing.

**TABLE 4:** Hypothesis tests for the genotype-by-SES interaction model.

Trait	Homogeneous additive genetic variance	Homogeneous residual environmental variance	Homogeneous household variance	Genetic correlation equals 1	Household correlation equals 1
Fasting glucose	0.0087	0.0301	-----	0.50000	-----
2-h glucose	0.00465	0.0237	-----	0.00575	-----
Total serum cholesterol	1.00000	1.00000	1.00000	0.50000	0.50000
Systolic blood pressure	1.00000	1.00000	0.0166	0.50000	0.50000
Diastolic blood pressure	1.00000	1.00000	1.00000	0.50000	0.50000

**FIGURE 1:**
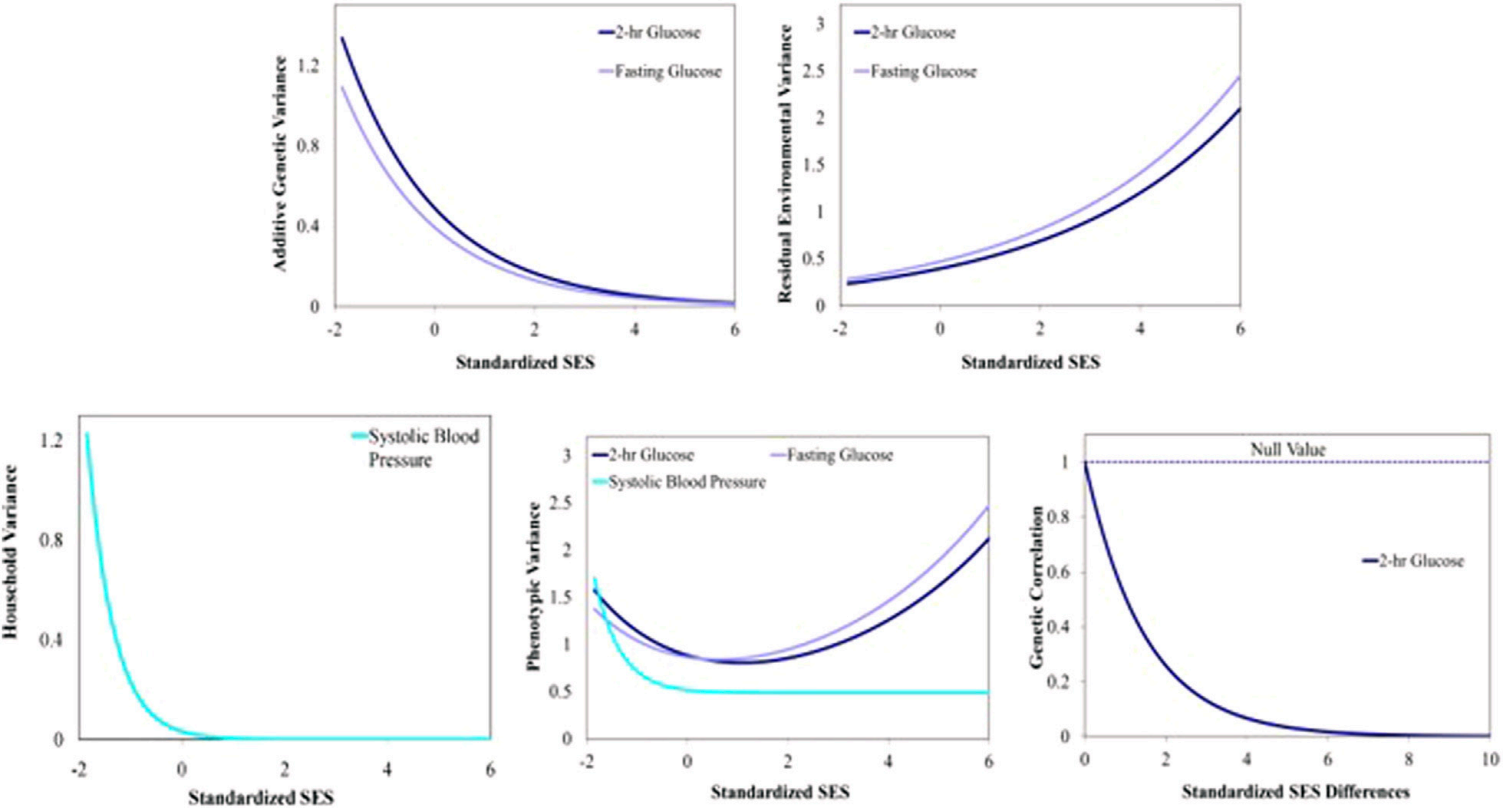
Variance and correlation functions for several Metabolic Syndrome traits. Top left: Additive genetic variance functions. Top right: Residual environmental variance functions. Bottom left: Household variance function. Bottom middle: Total phenotypic variance functions. Bottom right: Additive genetic correlation function.

Results of the specific GxE hypothesis tests and of the tests of homogeneous residual environmental variance, homogeneous household variance component, and $\rho_{c}=1$ are presented in Table 4. We found evidence of GxE interaction due to heterogeneity in additive genetic variance in fasting glucose and 2-h glucose (Table 4; Figure 1). We also found evidence of heterogeneity in residual environmental variance for fasting glucose and 2-h glucose (Table 4; Figure 1). Because additive genetic variance is decreasing while residual environmental variance is increasing with zSES for 2-h glucose and fasting glucose, total phenotypic variance exhibits a superficial quadratic-like behavior along the SES environment (Figure 1). We also found evidence of GxE due to genetic correlation being less than 1 for 2-h glucose (Table 4; Figure 1). Of the 3 traits (total serum cholesterol, systolic blood pressure, and diastolic blood pressure) that exhibited significant household effects and that progressed to this stage of the analyses, only systolic blood pressure showed significant heterogeneity in its household variance (Table 4; Figure 1). The household variance function for systolic blood pressure behaved in a similar manner to the additive genetic variance functions for fasting glucose and 2-h glucose. Given that the additive genetic and residual environmental variance functions for systolic blood pressure were constant (Table 4), the behavior of the phenotypic variance function was determined entirely by the household variance function, which explains their similarity (Figure 1).

**FIGURE 2:**
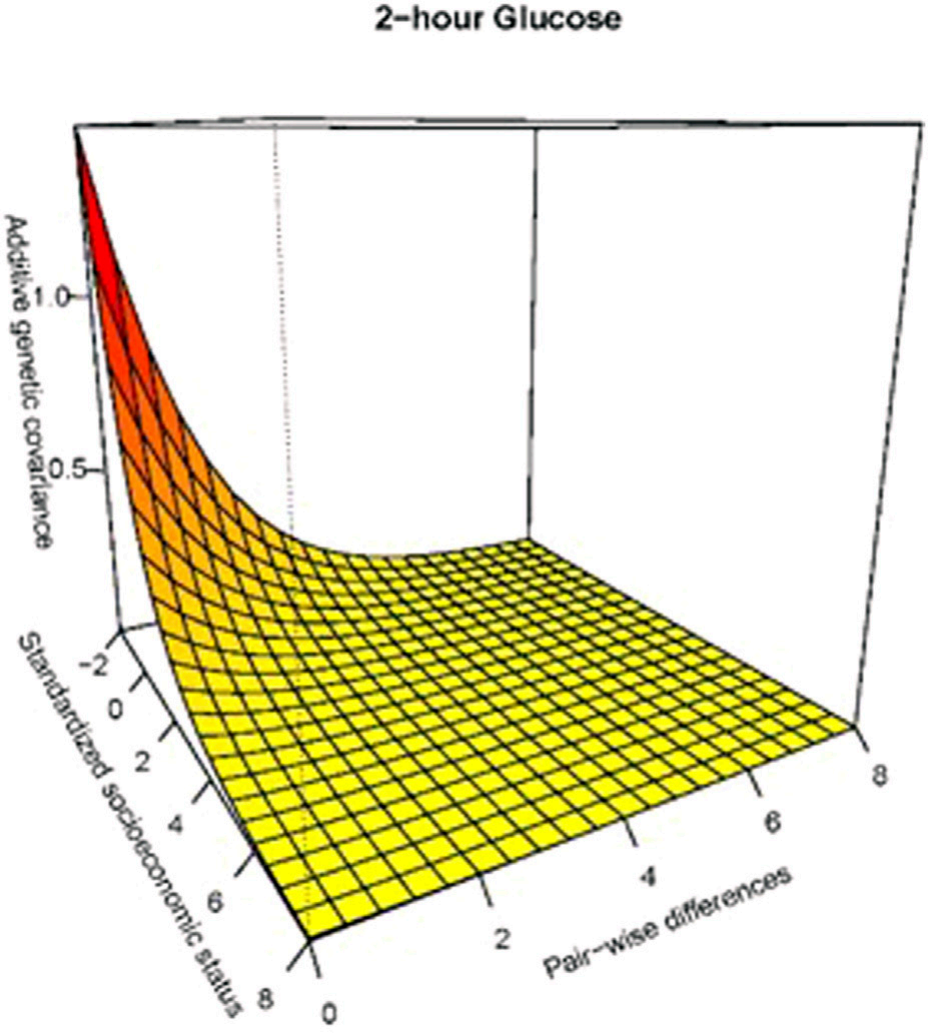
Additive genetic covariance function for 2-h glucose. Expressed joint behavior of the additive genetic variance and correlation functions in producing covariance heterogeneity representing additive genetic covariance at the lower SES level. Additive genetic covariance function for 2-h glucose. The additive genetic covariance function in the vertical axis is a joint function of the additive genetic variance displayed along the standardized socioeconomic status (zSES) axis and the genetic correlation function displayed along the pair-wise differences (in zSES values) axis.

In Figure 2, we express the joint behavior of the additive genetic variance and correlation functions in producing covariance heterogeneity for 2- hour glucose.

## Discussion

Our results are consistent with the epidemiological relationship between SES and MS risk factors in Latinos as well as other ethnic groups (Hostinar et al., 2017b; Engin, 2017; Blanquet et al., 2019). The clinical implications of the heritability of the 12 phenotypes studied imply that although the genetic components are moderate, the environmental influences are greater and complementary. Hispanic/Latinos exhibit an inverse association between SES and metabolic syndrome of varying magnitudes across SES variables. For the glucose variables, there was evidence of GxE via heterogeneity in additive genetic variance and we consistently observed that additive genetic variance was higher in the lower end of the SES spectrum (Figures 1, 2). The genetic effects underlying cardiovascular disease are similarly dynamically modulated along the SES spectrum (Schultz et al., 2018; Carter et al., 2019; Powell-Wiley et al., 2022; Diego et al., 2023).

To the general patterns regarding SES, mortality, and morbidity measures, we can add the inverse association between SES and additive genetic variance in 2-h glucose and fasting glucose. We found evidence of GxE for fasting glucose and 2-h glucose (Figure 2) that suggests that different sets of genes are active in different regions of the SES spectrum. These results indicate that the gene networks influencing glucose are upregulated in lower relative SES environments. Our results demonstrate that the SES environment modulates the underlying genetic architecture, and this interaction is related to glucose homeostasis (Puolakka et al., 2016; Corsi and Subramanian, 2019).

After accounting for sex effects before GxE analysis, the inverse association between SES and morbidity measures is still significantly more robust in women than in men among Mexican Americans from San Antonio (Stern et al., 1984). Accounting for behavioral variables, especially physical activity, attenuates the strength of the inverse relationship between SES and morbidity measures of MS (Walker et al., 2014; Tanaka et al., 2018).

There are important household effects for systolic blood pressure. The household variance function for systolic blood pressure was also inversely related to SES. In addition to the dynamic genetic effects discussed above, this is another novel twist to the general pattern of an inverse relationship between measures of MS traits and SES.

Our conclusions have real-world applicability in clinical practice and public health strategies. Addressing health disparities, especially in vulnerable populations like Mexican Americans in south Texas, should be a priority. Our findings highlight the importance of reducing health inequities by addressing both genetic and socioeconomic factors contributing to MS risk. By recognizing the interplay between genetics and SES in the development of metabolic syndrome, healthcare professionals and policymakers can work together to develop targeted interventions and policies that improve the health outcomes of communities, particularly those facing socioeconomic challenges, similar to the study population.

The identification of gene-environment interactions (GxE) related to metabolic syndrome (MS) risk factors suggests that healthcare interventions can be tailored based on an individual’s genetic profile and socioeconomic status (SES). Understanding that the genetic effects underlying MS traits are modulated by SES provides insights into targeting high-risk populations. Healthcare providers and public health initiatives can focus their efforts on communities with lower SES, as these individuals may have a higher genetic susceptibility to MS-related risk factors. Public health strategies should emphasize health education and promotion, especially in lower SES communities. This includes raising awareness about the importance of healthy lifestyle choices such as diet, especially in relationship to carbohydrates. Recognizing the inverse association between SES and additive genetic variance in glucose variables (fasting and 2-h glucose) suggests that individuals from lower SES backgrounds may benefit from earlier screening and detection of MS risk factors. This can help identify at-risk individuals and initiate preventive measures at an earlier stage.

Public health policies can be designed to address the social determinants of health that are linked to SES. Initiatives to improve access to education, reduce chronic generational poverty, enhance neighborhood environments, and promote healthy behaviors can have a positive impact on reducing MS risk. Healthcare providers should consider a patient’s SES as a vital factor in assessing their risk for metabolic syndrome. By incorporating SES into clinical practice, clinicians can provide more comprehensive and tailored care to individuals, considering both genetic and environmental factors.

## Implications for research

Our results support the presence of GxE interaction between genetic factors and SES in determining the risk for metabolic syndrome in Mexican Americans.

We are now further exploring the GxE interaction using mRNA sequencing data to identify the contribution of specific genes and canonical pathways.

### Limitations

As this is our initial report of genotype-by-environment interaction (GxE) in relation to SES from the Mexican American Family Study, we aimed first to establish that the SES environment is key by constructing a multivariate representation of it. We want to discuss the limitations of our study, including that we statistically combined the three SES components to obtain a composite “overall” SES variable. We anticipate pursuing more focused GEI studies per individual SES component in future investigations. The Duncan Socioeconomic Index uses occupations and information based on 1950 census data on non-Hispanic white, job-prestige data, and there are newer methods to calculate SES. Using specific income and education data in the population studied may provide more specific SES effects. Although our GxE findings are from older data and not specifically causal, other GWAS studies have demonstrated similar gene-based results. Certainly, changes over the last 3 decades in healthcare, lifestyle, and the environment have affected the health of patients with metabolic syndrome. It is not our intention to explain how the interaction occurs, but rather that genes underlying MS interact with SES. The important point is that risk factors for MS respond to SES. Future research is needed to determine if increased prevalence and sequalae of MS will respond differently to SES, but what we find important is that the interaction occurs at a molecular level.

## Conclusion

We present evidence for the dynamic modulation of the genetic architecture of major components of MS by the SES environment. These findings highlight the importance of SES environmental effects on health-related physiology. Our study serves as a foundation for further research into transcriptomic and methylomic analysis to identify the specific proteins, canonical pathways, and molecular changes involved in the GxE interaction. We then can explore interventions that mitigate the negative health impacts associated with low SES, such as targeted genetic therapies or lifestyle interventions.

## Data Availability

The raw data supporting the conclusions of this article will be made available by the authors, without undue reservation.
