# Supplementary figures and images for: Metabolic syndrome traits exhibit genotype-by-environment interaction in relation to socioeconomic status in the Mexican American family heart study
Source: Front Genet. 2024 Mar 1;15:1240462. doi: 10.3389/fgene.2024.1240462 (PMC10940335; doi:10.3389/fgene.2024.1240462)

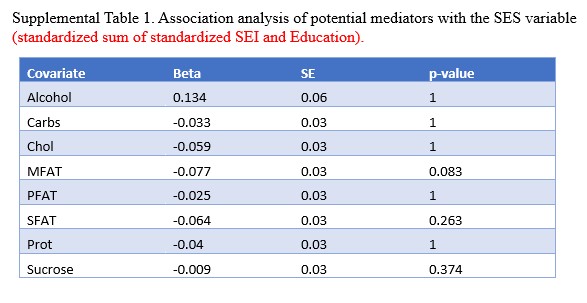

Supplement: Supplementary file 1 [file Image1.jpeg]
