# Supplementary material for: Metabolic syndrome traits exhibit genotype-by-environment interaction in relation to socioeconomic status in the Mexican American family heart study
Source: Front Genet. 2024 Mar 1;15:1240462. doi: 10.3389/fgene.2024.1240462 (PMC10940335; doi:10.3389/fgene.2024.1240462)
Supplement: Supplementary file 2 [file DataSheet1.PDF]

The following is a comprehensive background on the statistical methods used for analyzing genotype-by-environment (GxE) interactions including a detailed explanation of each statistical variable, its relevance, and significance in the context of GxE research.

Statistical Genetic Models

I. Polygenic model

For a generic phenotype vector $\mathbf{y}$ we write:

$$\mathbf{y} = \mathbf{X\beta} + \mathbf{g} + \mathbf{e}$$

where $\mathbf{X}$ is a matrix of covariates augmented at the left by a column of 1s, $\boldsymbol{\beta}$ is a vector of the intercept parameter and corresponding regression coefficients, and $\mathbf{g}$ and $\mathbf{e}$ are unobserved random genetic and environmental effects, respectively. The phenotypic covariance matrix, denoted by $\boldsymbol{\Sigma}$, is given as:

$$\boldsymbol{\Sigma} = \mathbf{K}\sigma_g^2 + \mathbf{I}\sigma_e^2,$$

where $\mathbf{K}$ and $\mathbf{I}$ respectively give genetic relationship and identity matrices, and σ_g^2 and σ_e^2 are correspondingly the additive genetic and environmental variance components. In statistical genetics, this base model is called the polygenic or additive genetic model. It will be instructive at this point to give the scalar phenotypic covariance equation specifying the elements of the covariance matrix for all possible ij-comparisons:

$$\sigma_p(y_i, y_j) = k_{ij}\sigma_g^2 + \delta_{ij}\sigma_e^2 = \begin{cases} \sigma_g^2 + \sigma_e^2 & \forall\ i = j; k_{ij} = 1; \delta_{ij} = 1 \\ k_{ij}\sigma_g^2 & \forall\ i \neq j; \delta_{ij} = 0 \end{cases}$$

where $\sigma_p(y_i, y_j)$ denotes the phenotypic covariance, k_{ij} is the ij-th element in $\mathbf{K}$, and $\delta_{ij} = \begin{cases} 1 \ \forall \ i = j \\ 0 \ \forall \ i \neq j \end{cases}$ denotes Kronecker delta. The top case on the right-hand side gives the diagonal elements of the covariance matrix under the polygenic model, and the bottom case gives the off-diagonal elements.

II. Modeling genotype-by-environment interaction for continuous environments

The polygenic model is used to obtain estimates of trait heritability, defined as the ratio of the additive genetic variance to the total phenotypic variance, $h^2 = \frac{\sigma_g^2}{\sigma_g^2 + \sigma_e^2} = \frac{\sigma_g^2}{\sigma_p^2}$, and as a model reference point upon which more complex models can be elaborated. For a sample of related individuals, assuming fully uncorrelated genetic and environmental effects, the polygenic model posits that the phenotypic covariance is decomposable into additive genetic and residual environmental variance components and that inter-individual covariances will be given strictly by the additive genetic variance weighted by the genetic relatedness coefficient (see the polygenic covariance equation). The latter feature of the polygenic model makes two implicit assumptions regarding the genetic covariance: that the pairwise genetic correlation is unity and that the additive genetic variance is homogeneous. Explicitly modeling these assumptions is key to our approach to modeling G×E interaction.

For the simplest case of contrasting two different environments, the G×E variance is zero if the following two conditions are simultaneously true: homogeneity in the additive genetic variance: $\sigma_{g1}^2 = \sigma_{g2}^2 = \sigma_g^2$, where σ_{g1}^2 and σ_{g2}^2 are the additive genetic variances in environments 1 and 2 (for example, males and females for a G×Sex model

or unaffecteds and affecteds for a G×dichotomous-disease model), respectively; the genetic correlation (ρ_g) is one across environments: $\rho_g = 1$. Denoting the G×E variance as $\sigma^2_{g\Delta}$, we have the expression:

$$\sigma^2_{g\Delta} = \begin{cases} \sigma^2_{g1} + \sigma^2_{g2} - 2\rho_g\sigma_{g1}\sigma_{g2} & \forall\ \sigma^2_{g1} \neq \sigma^2_{g2} \\ 2\sigma^2_g(1-\rho_g) & \forall\ \sigma^2_{g1} = \sigma^2_{g2} = \sigma^2_g \end{cases}$$

There is G×E evidence if either null hypothesis is rejected. Rejection of either or both is evidence that the phenotypic response to the environment has a genetic basis.

We can extend this theory to an environmental spectrum to model G×E for in relation to the continuous SES variable as opposed to two levels of the environmental variable. To this end, we employ variance and correlation functions, which we define as:

$$\sigma^2_g = exp\left[\alpha_g + \gamma_g zSES_i\right], \text{ and } \rho_g = exp\left(-\lambda_g\left|zSES_i - zSES_j\right|\right),$$

where the additive genetic variance is reparameterized as an exponential function of the $zSES$ variable for the ith individual, and where the genetic correlation is reparameterized as an exponential decay function of the difference of $zSES$ variables for any pair of individuals i and j, and where α_g, γ_g, and λ_g are parameters to be estimated. These functions can be interpreted as the variance and correlation functions of a Gaussian stationary stochastic process, where the index variable of the stochastic process is the SDHI environment. The statistical null hypotheses under the reparameterizations for variance homogeneity and genetic correlation stationarity at unity, respectively, are given as $\gamma_g = 0$ and $\lambda_g = 0$.

On the logarithmic scale this gives us a straightforward way to assess potential heterogeneity by way of the γ_g parameter which essentially becomes a slope term:

$$\ln \sigma_g^2 = \alpha_g + \gamma_g(zSES_i)$$

For $\gamma_g = 0$, we have the null hypothesis of variance homogeneity. For $\gamma_g > 0$ or $\gamma_g < 0$, the variance is increasing or decreasing as a function of the $zSES$ variable. Regarding the null hypothesis of a genetic correlation equal to 1, the exponential decay function is a rational choice because when $\lambda_g = 0$, we have that $\rho_g = e^0 = 1$. Moreover, the parameter λ_g gauges the extent to which the genetic correlation decays away from 1 as a function of differences in the $zSES$ variable. To guard against model misspecification bias, we also model the residual environmental variance as a function of the $zSES$ variable in the same way as the additive genetic variance. The phenotypic covariance function, with components given by the variance and covariance functions of a Gaussian stationary stochastic process, can be partitioned as follows:

$$\sigma_p(y_i, y_j) = \begin{cases} \sigma_g^2 + \sigma_e^2 & \forall\, i = j;\ \delta_{ij} = 1 \\ \sigma_i \sigma_j \rho_g & \forall\, i \neq j;\ \delta_{ij} = 0 \end{cases}$$

$$= \begin{cases} exp\left[\alpha_g + \gamma_g(zSES_i)\right] + exp[\alpha_e + \gamma_e(zSES_i)] & \forall\, i = j;\ \delta_{ij} = 1 \\ \left\{exp\left[\alpha_g + \gamma_g(zSES_i)\right]\right\}^{\frac{1}{2}} \left\{exp\left[\alpha_g + \gamma_g\left(zSES_j\right)\right]\right\}^{\frac{1}{2}} & \\ \qquad \cdots \times exp\left(-\lambda_g\left|zSES_i - zSES_j\right|\right) & \forall\, i \neq j;\ \delta_{ij} = 0 \end{cases}$$

$$= \begin{cases} exp\left[\alpha_g + \gamma_g(zSES_i)\right] + exp[\alpha_e + \gamma_e(zSES_i)] & \forall\, i = j;\ \delta_{ij} = 1 \\ exp\left[\alpha_g + \frac{1}{2}\gamma_g\left(zSES_i + zSES_j\right) - \lambda_g\left|zSES_i - zSES_j\right|\right] & \forall\, i \neq j;\ \delta_{ij} = 0 \end{cases}$$

We define a genetic covariance matrix $\mathbf{\Psi} = \{\psi_{ij}\}$, with elements given as follows:

$$\psi_{ij} = \begin{cases} exp\left[\alpha_g + \gamma_g(zSES_i)\right] & \forall\, i = j;\;\; \delta_{ij} = 1 \\ exp\left[\alpha_g + \frac{1}{2}\gamma_g\left(zSES_i + zSES_j\right) - \lambda_g\left|zSES_i - zSES_j\right|\right] & \forall\, i \neq j;\;\; \delta_{ij} = 0 \end{cases}$$

We posit a diagonal matrix $\mathbf{\Omega} = diag\{\omega_{ii}\}$ with diagonal elements containing the residual environmental variance function, $\omega_{ii} = exp[\alpha_e + \gamma_e(zSES_i)]$. The covariance matrix for this G×E model for continuous environments is then given as follows:

$$\mathbf{\Sigma} = \mathbf{K} \odot \mathbf{\Psi} + \mathbf{\Omega}$$

where $\odot$ is the Hadamard matrix-multiplication operator (element-wise multiplication).
